# Drug Delivery System Based on Carboxymethyl Cellulose Containing Metal-Organic Framework and Its Evaluation for Antibacterial Activity

**DOI:** 10.3390/polym14183815

**Published:** 2022-09-12

**Authors:** Fatimah A. T. Alsaaed, Hany M. Abd El-Lateef, Mai M. Khalaf, Ibrahim M. A. Mohamed, Mohammed A. Al-Omair, Mohamed Gouda

**Affiliations:** 1 Department of Chemistry, College of Science, King Faisal University, Al-Ahsa 31982, Saudi Arabia; 2 Chemistry Department, Faculty of Science, Sohag University, Sohag 82524, Egypt

**Keywords:** drug delivery, nanocomposites, antibacterial activity, metal–organic framework

## Abstract

A novel drug delivery system based on carboxymethyl cellulose containing copper oxide at melamine and zinc oxide at melamine framework (CMC-Cu-MEL and CMC-Zn-MEL) was prepared by the hydrothermal route. Synthesized nanocomposites were characterized by FTIR, SEM, and Raman spectroscopy. In addition, transmission electron microscopy (TEM) and selected area electron diffraction (SAED) images were applied to confirm the particle size and diffraction pattern of the prepared nanocomposites. Furthermore, the crystallinity of the synthesized CMC, CMC-Cu-MEL, and CMC-Zn-MEL materials was studied via X-ray diffraction (XRD). Estimating the transport exponent, which discloses the solvent diffusion and chain relaxation processes, and the Ritger–Peppas kinetic model theory were used to control the TC release mechanism from CMC-Cu-MEL and CMC-Zn-MEL. Additionally, the CMC-Cu-MEL and CMC-Zn-MEL containing TC had the highest activity index percents of 99 and 106% against S. aureus and 93 and 99% against *E. coli*, respectively. The tailored CMC-Cu-MEL and CMC-Zn-MEL for drug delivery systems are expected to be feasible and efficient.

## 1. Introduction

Various novel strategies for creating drug delivery systems have been developed in recent years [1,2,3]. The optimum drug delivery method should provide an adequate concentration of medication to the target areas while also improving treatment effectiveness [4,5]. Finding a good delivery carrier is thus one of the primary problems in achieving this goal [6,7]. Polymeric particles [8,9], nanomaterials [7,10], microspheres [11], dendrimers [12], and liposomes [13] have all been employed as possible drug carriers; however, nanomaterials [7], microspheres [11], dendrimers [12], and liposomes [13] have all shown benefits for drug delivery [14] in addition to energy applications [15] or water treatment devices [16,17]. Nanoparticle-based therapies can improve the efficacy of conventional medications and help to avoid many of the free drug therapeutic problems. Nanocarriers can significantly increase the efficacy and distribution of medications, particularly those that have low water-solubility [18].

Nanoparticle drug conjugates were produced in clinical therapy at various stages, demonstrating the clinical effectiveness of nanoparticle therapeutics [5,19]. The synthesis of metal–organic framework (MOF) and carboxymethyl cellulose (CMC) nanocomposites has recently been reported in various publications [20,21,22,23,24]. An MOF consists of organic linkers and metal ions as nodes that self-assemble [21,23,25,26]. The concept of MOF nanocomposites was established because CMC comprises carboxylic and hydroxyl groups that allow the action of metal ions in MOFs to be generated as a composite [22].

One of the cellulose derivatives, carboxymethyl cellulose (CMC), for example, is an anionic water-soluble biopolymer [27,28,29,30]. CMC has distinctive properties, such as pH sensitivity, hydrophilicity, nontoxicity, biodegradability, and biocompatibility [31,32,33]. Because of these CMC properties, it is used as a carrier that has sparked much interest in biological applications [9,34,35]. As a result, CMC may be a promising candidate for nanoparticle modification [36,37].

Tetracycline (TC) is an antibiotic that is widely used in the industry to treat a variety of illnesses. Acne, cholera, plague, malaria, brucellosis, and syphilis are examples [38]. Irritation of the gastrointestinal area (GIA) is one of the most common adverse effects seen after taking TC orally. The medicine can be encapsulated into biocompatible support as enteric-coated dosage forms to reduce the high GIA adverse effects induced by the oral usage of TC. Furthermore, nanomaterial supports with a large surface area, such as MOFs, can increase medicinal effectiveness, and medical enactment can be enriched even further by adjusting nanoparticles.

The aim of this work was to find a novel low-cost drug delivery system based on carboxymethyl cellulose containing copper oxide melamine and a zinc oxide melamine framework (CMC-Cu-MEL and CMC-Zn-MEL) via a simple technique (hydrothermal route). These nanocomposite materials were characterized by various physicochemical techniques to indicate the incorporation of transition metal oxide into the CMC polymer content. Tetracycline hydrochloride was loaded into the prepared nanocomposites and its release mechanism was examined in PBS. The antibacterial activities of the prepared samples were evaluated against *S. aureus* and against *E. coli*.

## 2. Materials and Methods

### 2.1. Materials

Metal salts, zinc nitrate hexahydrate [Zn(NO_3_)_2_-6H_2_O,98%] and copper nitrate trihydrate (Cu(NO_3_)_2_-3H_2_O), were purchased from BDH Chemicals, dimethyl sulfoxide (DMSO) (99.8%) was purchased from Loba Chemie (LOBA CHEMIE PVT. LTD, Jehangir Villa, Mumbai, INDIA, cellulose powder from spruce was prepared in Fluka AG Laboratories (Buchs, Switzerland), sulfuric acid (95–97%) and 2-propanol (99.8%) were purchased from Sigma-Aldrich (St. Louis, MO, United States, formaldehyde, 2,4,6-triamine-1,3,5-triazine, 99% (Melamine) was purchased from Sigma-Aldrich (St. Louis, MO, United States).

### 2.2. Synthesis of Carboxymethyl Cellulose (CMC)

The carboxymethyl cellulose (CMC) sample was prepared to utilize the alkalization and carboxymethylation processes in accordance with the stated method [39]. An alkalization process was produced by the 2:1 molar ratio of sodium hydroxide to cellulose in the presence of 50/50 (*v*/*v*) water and isopropanol, which was stirred mechanically for 10 min. Monochloroacetic acid (1 mol) was then added to the mixture and blended for 5 min with a mechanical blender. After that, it was transferred to a glass bottle before spending 3 h in an 80 °C water bath. To eliminate unreacted sodium hydroxide and monochloroacetic acid, prepared samples were washed with water/isopropanol combination 50:50 (*v*/*v*) for 48 h at 70 °C. The produced sample was then baked at 70 °C to test how much carboxyl it contained. The prepared samples had a carboxyl content of 62 meq./100 g sample, which is equivalent to a 0.58 degree of substitution. The degree of substitution was calculated according to carboxyl content, which was determined by the acid–base titration method using the following equations:

(1) $$n\;COOH=\frac{\left(V1-V2\right)\;\times \;conn.\;of\;HCl}{w}$$

where *nCOOH* is the carboxyl content, *V*1 is the volume of NaOH, *V*2 is the volume of HCl, and w is the dry weight of prepared sample

(2) $$DS=\frac{162\;\times \;nCOOH}{w-58\;\times \;nCOOH}$$

where 162 is the molecular weight of unhydroglucose unit and 58 is the molecular weight of *COO*.

### 2.3. Synthesis of Carboxymethyl Cellulose Containing Cu-melamine and Zn-melamine Framework

The hydrothermal method of aqueous Zn and Cu was used to produce ZnO and CuO/CMC nanocomposites, and the methods followed those described in the literature [40]. To make a diluted colloidal solution (0.5 wt percent), CMC (5.0 g) was suspended in distilled water and then sonicated for 10 min to scatter the CMC. Then, with the mixture continuously stirred in a nitrogen atmosphere, 1.0 mg of citric acid, 1.0 mL of 2 mole/L HCl, and 1.3 mmoles of metal salt (Zn(NO_3_)_2_-6H_2_O and Cu(NO_3_)_2_-3H_2_O) were added. Dropwise addition of 10 mL of a 2 mole/L NaOH solution was added to the aforementioned solution, followed by 120 min of stirring at 25 °C while under nitrogen at 500 rpm. The produced CMC/ZnO and CMC/CuO composites were centrifuged at 15,000 rpm for 15 min after being cleaned and separated many times with distilled water. Melamine formaldehyde and prepared CMC/ZnO and CMC/CuO nanocomposites were dispersed in ethanol and subjected to a 10 min sonication process. CMC/ZnO and CMC/CuO nanocomposites were added with a mechanical stirrer to melamine formaldehyde ethanol solution, and the mixture was stirred continuously for four hours. The CMC-Cu-MEL and CMC-Zn-MEL nanocomposites were then separated by centrifugation at 6000 rpm for 10 min, dried at 50 °C under vacuum, and washed numerous times with a water/ethanol mixture (50:50). Additionally, a stainless steel boat containing the mixed nanocomposite samples was transported to and placed in a muffle furnace at 250 °C for five hours [41]. Using a centrifuge set at 10,000 rpm for 10 min, cured samples were first rinsed several times with a water/ethanol solution before being dried for five hours at 65 °C using a vacuum drier. The preparation mechanism started with metal/CMC interaction in the presence of a strong alkaline medium to form the oxide form of the Cu or Zn at the CMC polymer. After that, physical contact by sonication and stirring between melamine and oxide/polymer was done to confirm the homogeneity of the prepared composite. Finally, the formation of CMC-Zn-MEL or CMC-Cu-MEL was carried out by maintaining the obtained solid at 250 °C for five hours. During this high temperature, melamine interacted with the oxide/CMC to form CMC-Zn-MEL or CMC-Cu-MEL.

### 2.4. Characterization Techniques

The functional groups in the samples were identified using a Fourier transform infrared (FTIR) spectrometer (Spectrometer Model FTIR-8400S, Shimadzu, Japan) in the range 400.0 to 4000.0 cm^−1^. A scanning electron microscopy (SEM, Joel Jsm6360LA, Tokyo, Japan) was used to observe the morphology of the synthesized CMC, CMC-Cu-MEL, and CMC-Zn-MEL materials. Additionally, a transmission electron microscope (TEM) in a Jeol-1230 electron microscope was used to affirm the size of the prepared particles of CMC, CMC-Cu-MEL, and CMC-Zn-MEL materials. The crystallinity of the synthesized CMC, CMC-Cu-MEL, and CMC-Zn-MEL materials was studied via X-ray diffraction (XRD; TD-3500 diffractometer, Dandong Mastery Technology Co., Ltd, Dandong, China) at room conditions with Ni-filtered CuKα radiation (λ = 1.5418° A), at 40 kV and 30 mA. Raman spectroscopy was utilized to study the stability of the chemical structure of the prepared CMC, CMC-Cu-MEL, and CMC-Zn-MEL materials using a Raman spectrometer (Horiba Scientific, Unit D Fletcher, CA, USA). The nitrogen adsorption–desorption isotherm was utilized to determine the impact of Cu and Zn oxide incorporation on the CMC matrix by using the Brunauer–Emmett–Teller (BET) method at 77 K (Tristar II 3020 version 3.02, Norcross, GA, USA).

### 2.5. Preparation of TC-Loaded CMC, CMC-Cu-MEL, and TC-Loaded CMC-Zn-MEL

Tetracycline hydrochloride (TC) loading in the CMC-Cu-MEL and CMC-Zn-MEL was carried out using the following method: The prepared CMC-Cu-MEL and CMC-Zn-MEL were impregnated in TC aqueous solution (0.1 mMole) and stored at room temperature for 72 h. The loaded samples were removed from the mixture using a centrifuge at 3000 rpm. The TC-loaded samples were washed several times with deionized water and centrifuged. The TC-loaded samples were dried in the vacuum oven. A UV–visible spectrophotometer at (*λ*_max_ = 360 nm) was used to confirm the presence of TC in CMC-Cu-MEL and CMC-Zn-MEL via determination of the free amount of TC from the impregnation. The TC-loaded CMC-Cu-MEL and TC-loaded CMC-Zn-MEL were 0.36 and 0.3 (mg/L), respectively.

### 2.6. Release Study

According to the described approach, UV–visible (UV-Vis-NIR UV 3600-spectrometer Shimadzu Company, Kyoto, Japan) absorbance is considered to indicate the release of TC from CMC-Cu-MEL and CMC-Zn-MEL nanocomposites [42] as follows: Each vessel holding the TC-loaded nanocomposite was doped with 50 mL of buffer saline phosphate (pH = 7.40), and the vessels were then incubated at 310 K. Each vessel holding the TC-loaded nanocomposite was doped with 50 mL of buffer saline phosphate (pH = 7.40), and the vessels were then incubated at 310 K. The CMC-Cu-MEL and CMC-Zn-MEL nanocomposites were dispersed individually in 0.2 g of the buffer solution to conduct release studies. To measure the absorbance using UV–visible spectroscopy at max = 360 nm, 3 mL methanol and 2 mL distilled water were mixed and added to each buffer. The concentration of the free TC was determined using a common calibration curve. Based on the findings, up to 24 h of data were plotted.

### 2.7. Antibacterial Activity

According to the published procedure, an agar well diffusion assay was used to test the antibacterial activity of CMC-Cu-MEL, CMC-Zn-MEL, and tetracycline hydrochloride against Gram-positive *Escherichia coli* and Gram-negative *Staphylococcus aureus* bacteria [42]. The antibacterial strains were planted on Petri dishes with 20.0 g of the dextrose agar nutrient, 5.0 g peptone, and 3.0 g of the beef extract. In DMSO, a nanocomposite containing 1.0 mM was created. Paper discs of a standard size (5.0 cm) were autoclaved and sterilized. The nanocomposite was then diluted by 20.0 L and added to the paper discs inside the Petri dishes. As a standard, the antibacterial medication ampicillin was mentioned. The plates were kept at 30 °C for one day of incubation. According to Equation (1), the clear zones (measured in mm) and activity index percentage were computed. The supporting documents include pictures of the Petri dishes.

(3) $$Activity\;Index\;\%=\frac{clear\;inhibition\;zone\;diameter\;of\;prepared\;samples\;}{clear\;inhibition\;zone\;diameter\;of\;ampicillin}\times 100$$

## 3. Results and Discussion

### 3.1. Morphology Investigation

**Figure 1:**
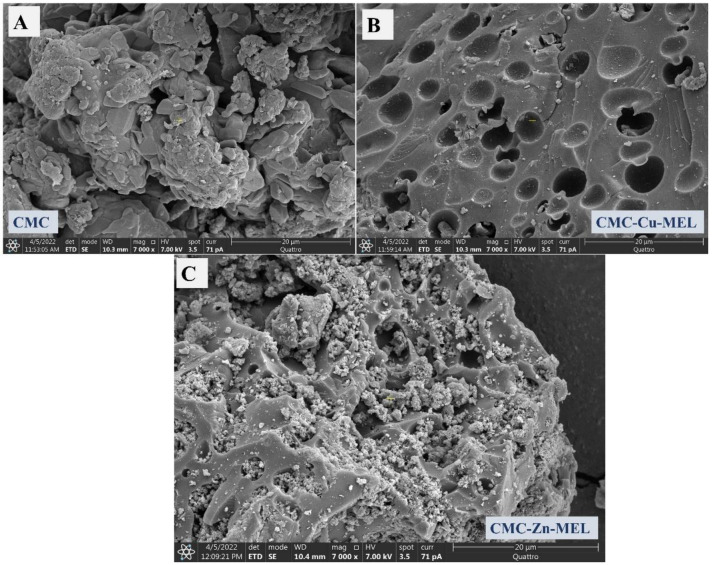
(**A**) SEM image of CMC, (**B**) SEM image of CMC-Cu-MEL, (**C**) SEM image of CMC-Zn-MEL.

**Figure 2:**
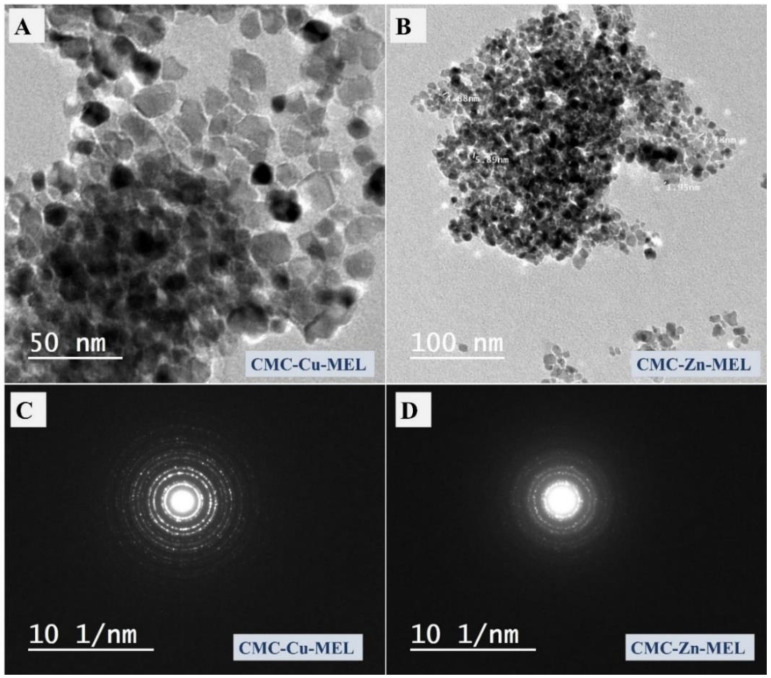
(**A**) TEM image of CMC-Cu-MEL, (**B**) TEM image of CMC-Zn-MEL, (**C**) SAED image of CMC-Cu-MEL, (**D**) SAED image of CMC-Zn-MEL.

**Figure 3:**
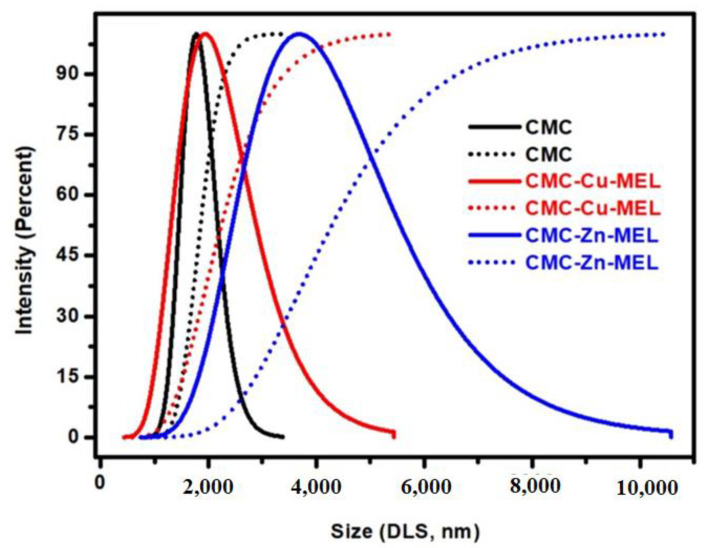
DLS analysis of CMC, CMC-Cu-MEL, and CMC-Zn-MEL.

The SEM and TEM images were utilized to investigate the morphology of the prepared CMC, CMC-Cu-MEL, and CMC-Zn-MEL, as shown in Figure 1 and Figure 2 for SEM, and TEM, respectively. For SEM images, the CMC one is shown in Figure 1A and has organic block behavior without isolated microscale or nanoscale particles. After that, CMC-Cu-MEL and CMC-Zn-MEL materials were additionally scanned and displayed in Figure 1B,C, respectively. The porosity was strongly increased in the case of CMC-Cu-MEL and small particles at the surface of the studied area as clarified in the blue circle of Figure 1C. The SEM images of CMC-Cu-MEL and CMC-Zn-MEL have heterogeneous characters with variable scale size particles, which indicate the impact of Cu and Zn on the morphology of CMC. The scale size of the synthesized particles of CMC-Cu-MEL and CMC-Zn-MEL was studied via TEM images, as shown in Figure 2A,B, respectively. The scale size was found to be between 5 and 25 nm, which confirms the nanoscale size of the prepared CMC-Cu-MEL and CMC-Zn-MEL materials. The traditional agglomeration of particles could not be observed in either the CMC-Cu-MEL or CMC-Zn-MEL materials. The SAED images of CMC-Cu-MEL and CMC-Zn-MEL are described in Figure 2C,D, respectively. These images have different ring patterns with variable diameters, which are assigned to different planes of the formed crystals. Additionally, these clear rings affirm the design of material in nanocrystalline characteristics. Therefore, the TEM, together with the SEM images, indicate that the prepared CMC-Cu-MEL and CMC-Zn-MEL materials have a heterogeneous surface containing small-size particles. Additionally, the prepared CMC, CMC-Cu-MEL, and CMC-Zn-MEL materials were studied by the dynamic light scattering (DLS) to confirm the particle size distribution (Figure 3). The size of the prepared particles by the DLS is larger than that previously seen via TEM because DLS analysis measures the hydrodynamic diameter. The highest intensity or frequency particle size was found at 1741.1, 1751.3, and 3594.1 nm for CMC, CMC-Cu-MEL, and CMC-Zn-MEL samples, respectively. The larger particle size was found in the CMC-Zn-MEL sample if compared with CMC-Cu-MEL. This trend could be due to filling some pores in the CMC matrix with crystalline metal Cu or Zn oxide. In short, DLS analysis indicates Cu- or Zn-loading to form CMC-Cu-MEL and CMC-Zn-MEL materials. The SEM image of CMC-Cu-MEL has more pores than CMC-Zn-MEL, which can be interpreted to indicate the smaller sample particle size of CMC-Cu-MEL compared to CMC-Zn-MEL.

### 3.2. Crystallinity Analysis

**Figure 4:**
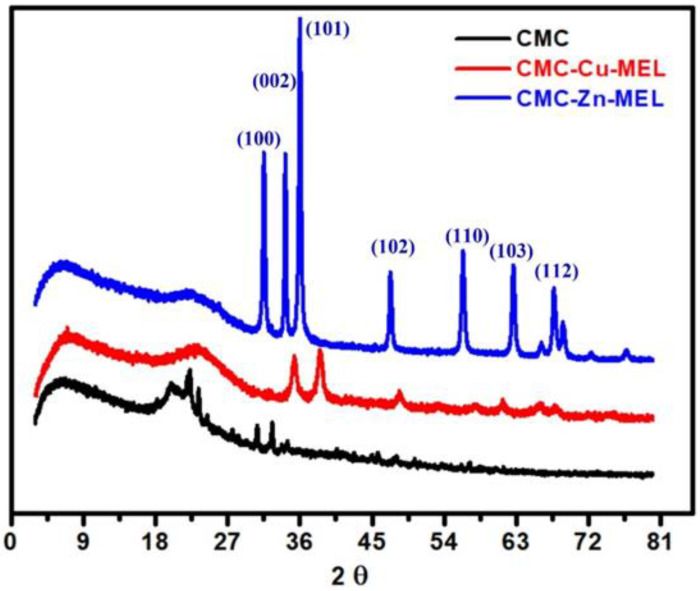
XRD analysis of CMC, CMC-Cu-MEL, and CMC-Zn-MEL.

XRD analysis confirmed the crystallinity of the CMC-Cu-MEL and CMC-Zn-MEL materials, as shown in Figure 4. The intensity of the diffraction peaks was sharply decreased after Cu or Zn incorporation. CMC XRD has an amorphous character with low-intensity XRD peaks at 2θ around 19.98, 22.34, 23.42, 30.62, and 32.58°. The appearance of these peaks as a broad peak apart from other high-intensity peaks in the case of CMC-Cu-MEL and CMC-Zn-MEL materials suggests the presence of ZnO or Cu oxide as filler in the polymer matrix of CMC. For CMC-Cu-MEL, the higher intensity peaks (more than pure CMC) were found at 35.26 and 38.66°, which indicate the formation of CuO as JCPDS 05–0661 [43]. The ZnO XRD peaks in the XRD analysis of CMC-Zn-MEL were observed at 2θ at 31.5, 34.18, 36.02, 47.32, 56.31, 62.66, and 67.64°. These peaks indicate the formation of ZnO in CMC-Zn-MEL as hexagonal wurtzite (JCPDS 36–1451) [44]. These peaks can be assigned to the following planes: (100), (002), (101), (102), (110), (103), and (112), respectively. Based on XRD analysis, it is proposed that CuO and ZnO were successfully incorporated into the CMC matrix.

### 3.3. Surface Area Analysis

**Figure 5:**
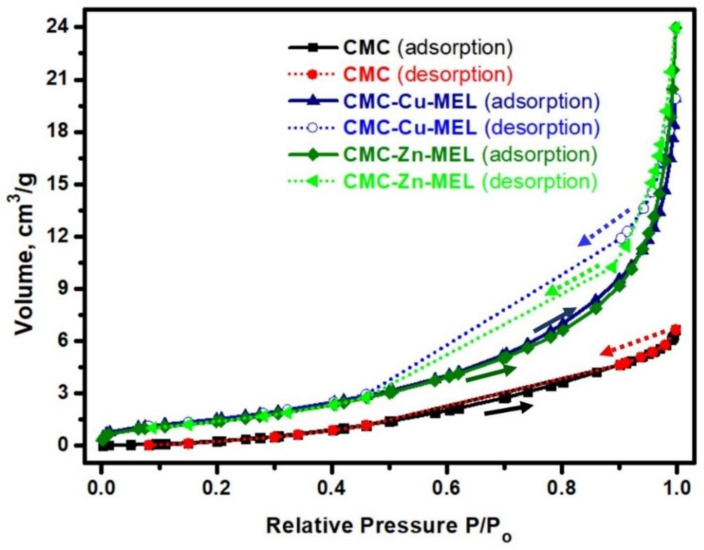
Nitrogen adsorption–desorption analysis of CMC, CMC-Cu-MEL, and CMC-Zn-MEL.

The surface area of the studied CMC, CMC-Cu-MEL, and CMC-Zn-MEL materials was investigated at 77 K via the nitrogen adsorption–desorption isotherm, as displayed in Figure 5. The adsorption curve of all CMC derivatives did not completely coincide with the desorption curve, and the isotherm behavior is near to a type (IV) isotherm, which is typical for mesoporous materials [45]. The BET surface area of CMC, CMC-Cu-MEL, and CMC-Zn-MEL materials were calculated and found at 64.4218, 5.8830, and 5.3964 m^2^/g, respectively. Therefore, the introduction of metallic parts decreased the estimated BET surface area. This trend could be due to filling some pores in the CMC matrix with crystalline metal Cu or Zn oxide. Although the surface area of CMC was less, the pores were filled with crystalline oxide that might create active sites for enhancement of crystallinity and performance. In short, BET analysis indicated the Cu- or Zn-loading to form CMC-Cu-MEL and CMC-Zn-MEL materials. The SEM image of CMC-Cu-MEL at high magnification reveals more pores than CMC-Zn-MEL, which can interpreted by the higher surface area of the CMC-Cu-MEL sample compared to the CMC-Zn-MEL sample. The absence of pores in the case of the CMC-Zn-MEL sample could be due to the larger size of the Zn radius (139 pm) compared to the Cu radius (128 pm). Therefore, the larger element (Zn) could fill more pores than that of the smaller element (Cu).

### 3.4. FTIR Analysis

**Figure 6:**
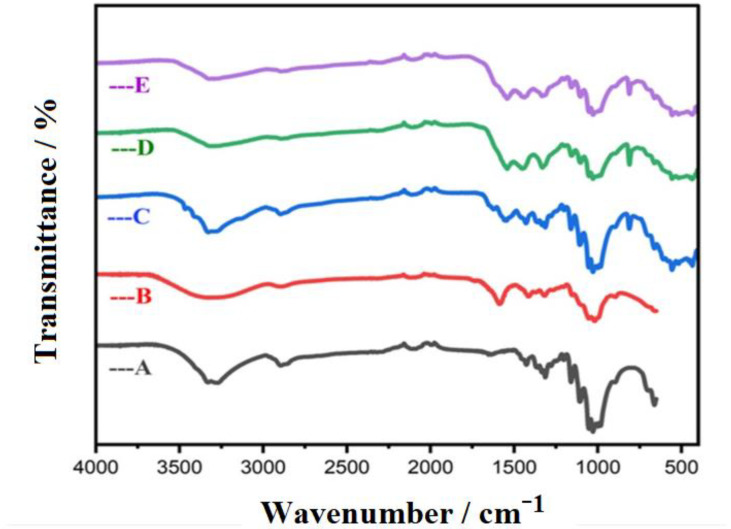
FTIR spectra for (**A**) cellulose, (**B**) CMC, (**C**) CMC/Melamine, (**D**) CMC/Melamine/Cu, and (**E**) CMC/Melamine/Zn composite.

FTIR analysis was used to investigate the chemical bonds or functional groups of the prepared CMC-Cu-MEL and CMC-Zn-MEL materials; in addition, pristine cellulose, CMC, and CMC-MEL were also studied (Figure 6). The stretching vibration of O-H groups around 3440 cm^−1^ was seen as a clear broad peak in the case of cellulose or CMC, or CMC-MEL, and as a small peak after Cu or Zn incorporation, which could be attributed to the expected interaction between the transition element (Cu or Zn) and the CMC oxygenated groups. The FTIR peak of symmetric C-H was observed at 2900 cm^−1^ with smaller intensity in the case of CMC-Cu-MEL and CMC-Zn-MEL materials [46,47]. The asymmetric vibrations of C-O-C were seen at 1160 cm^−1^ in all the samples [40]. Furthermore, the 1740 and 1180 cm^−1^ peaks could be assigned to C=O stretching and C-O-C stretching vibration, respectively [47,48]. The FTIR spectrum of the CMC-Cu-MEL and CMC-Zn-MEL materials has a peak at around 700 cm^−1^, which could correspond to M–O peaks (M = Cu or Zn) [48,49]. In short, the FTIR analysis indicates the successful incorporation of Cu or Zn in addition to the existence of most CMC function groups with lower transmittance.

### 3.5. Raman Spectroscopy Analysis

**Figure 7:**
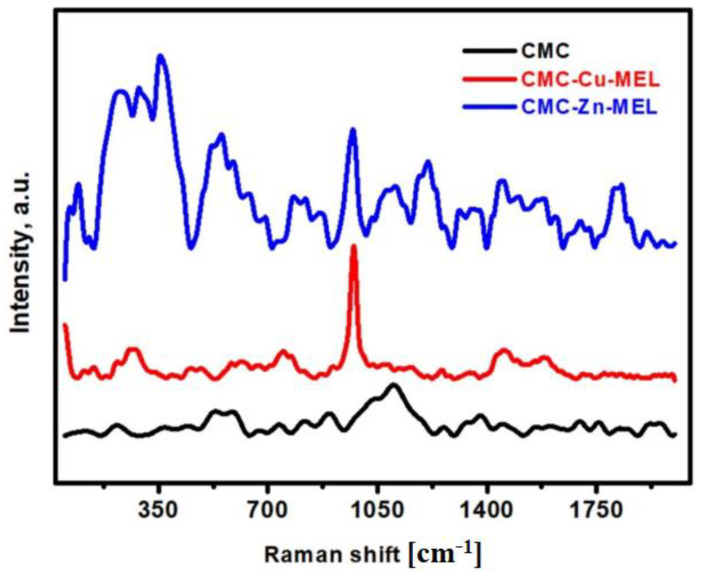
Raman spectroscopy analysis of CMC, CMC-Cu-MEL, and CMC-Zn-MEL.

Raman spectroscopy of the CMC, CMC-Cu-MEL, and CMC-Zn-MEL materials was investigated, as shown in Figure 7, in the range 50–2000 cm^−1^ for all studied samples extending vibrations. The main broad peak in the case of CMC without oxides at ~1095 cm^−1^ could be assigned to the amorphous C-O-C of the CMC polymer [50]. This peak declined after Cu or Zn-oxide incorporation, which indicates the interaction between CMC and Cu-oxide or Zn-oxide. The other bonds of CMC such as CCC, OCC, and OCO were observed in the range 150–600 cm^−1^ [40]. After oxide incorporation, there are three broad and clear peaks at 354, 556, and 970 cm^−1^, which were found in both the CMC-Cu-MEL and CMC-Zn-MEL samples. Typically, there are nine zones expected that are focused optical phonon modes (4A_u_ + 5B_u_ + A_g_ + 2B_g_), but three from these nine modes (A_g_ + 2B_g_) are Raman active. The first observed peak at 354^−1^, which could be related to the A_g_ mode, and the other peaks, evident at 556 and 970 cm^−1^,were related to B_g_ modes [40]. Briefly, Raman spectroscopy indicates the existence of Raman active vibration modes of oxide and polymer bonds.

### 3.6. TC Release Study

**Figure 8:**
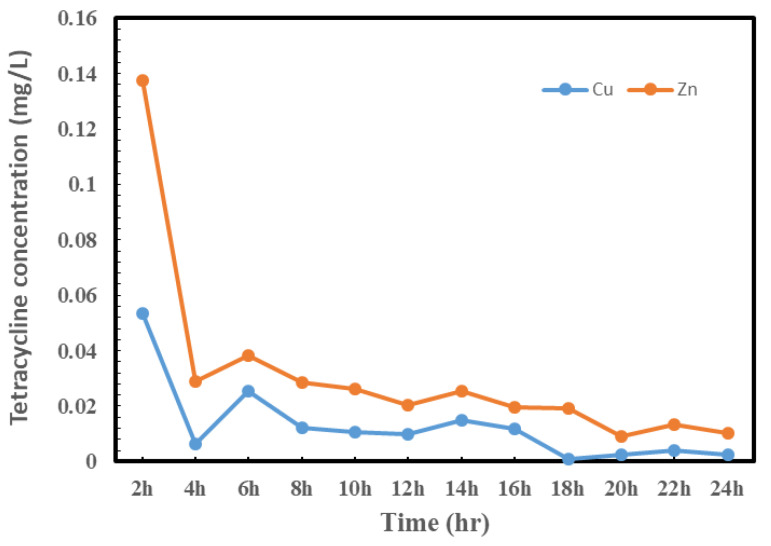
Release study of TC from CMC-Cu-MEL and CMC-Zn-MEL.

Metal–organic frameworks (MOFs) are routinely regulated in quantity form via a local transit method. As a result, the drug release only occurs at the desired location, preventing any systematic drug interaction. By using MOFs at the vulnerable position through destructive or nondestructive revenue, the longitudinal management of drug bearing can be quickly assumed. The rate of drug dissolution, drug dispersion, MOF size, drug physical desorption, and/or MOF degradation/erosion rates have recently been used to predict the order of drug release from MOFs [51]. The release of loaded TC from CMC-Cu-MEL and CMC-Zn-MEL in phosphate-buffered saline is shown in Figure 8. The data show that, due to the nature of burst release, the drug release rose for the first two hours and thereafter dropped. The burst release behavior during the first two hours was due to the presence of some TC over the surface of CMC-Cu-MEL and CMC-Zn-MEL, and the release that was exclusively from the CMC-Cu-MEL and CMC-Zn-MEL produced the difference in delivery behavior [52].

The Ritger–Peppas equation was used to study the release data for loaded TC from CMC-Cu-MEL and CMC-Zn-MEL (0.3%) within 24 h in order to fully comprehend the drug release process. This formula is displayed as Equation (2) [53]:

(4) $$\frac{M_{t}}{M_{\infty }}=kt^{n}$$

where the portion of TC released at time t is represented by $\frac{M_{t}}{M_{\infty }}$, and the parameters *k* (rate constant) and the exponent *n* were obtained by calculating the undeviating relation of log (*M*_*t*_/*M*∞) against log time (hour), which could afford evidence on the important release mechanisms. The intercept and slope values were determined when the relationship was plotted, and the transport exponent n was estimated to be approximately 0.32 at an R^2^ value of 0.966. The value of n falls between 0.2 and 0.8, taking into account the irregularity of the release mechanism (non-Fickian diffusion, which is a mixture of diffusion and polymer chain relaxation). The TC is then said to be released as a result of the polymeric chains relaxing and the solvent diffusing through them. This is due to the overwhelming influence of the diffusion process competing with the influence of the relaxation process [54].

### 3.7. Antibacterial Activity

The antibacterial evaluation of the synthesized TC-loaded CMC-Cu-MEL and TC-loaded CMC-Zn-MEL was our main concern. In this case, the antibacterial activity of the TC-loaded-CMC-Cu-MEL and TC-loaded-CMC-Zn-MEL were tested against Gram-positive *Staphylococcus aureus* and Gram-negative *Escherichia coli* bacteria. Table 1 and Figure S1 provide the corresponding clear areas of the inhibitory zones of the CMC-Cu-MEL and CMC-Zn-MEL and TC-loaded CMC-Cu-MEL and TC-loaded CMC-Zn-MEL samples. According to the data, CMC-Cu-MEL and CMC-Zn-MEL generally exhibited antibacterial activity against *S. aureus* as opposed to *E. coli* because of the presence of Cu and Zn, which had obvious inhibition zones with diameters of 6 mm and 12 mm, respectively. The latter is most likely caused by the bacteria’s cell wall’s variable selective permeability. Since *Escherichia coli* bacteria have two cytoplasmic membranes, which impede the drug’s penetration across its lipid membrane into the cytoplasm, *Staphylococcus aureus* exhibits stronger antibacterial efficacy than *Escherichia coli*. However, *Staphylococcus aureus* only has a thin cytoplasmic membrane and no outer lipid barrier, which facilitates better medication penetration. The antibacterial activity of the TC-loaded CMC-Cu-MEL and the TC-loaded CMC-Zn-MEL is also higher than that of the synthetic CMC-Cu-MEL and CMC-Zn-MEL. Synthesized TC-loaded CMC-Cu-MEL and TC-loaded CMC-Zn-MEL had the strongest antibacterial effects, with activity indices of 99 and 106 percent against *S. aureus* and 93 and 99 percent against *E. coli*, respectively. The synthesized CMC-Cu-MEL and CMC-Zn-MEL, on the other hand, showed very modest antibacterial action, with activity index percentages of 34 and 66 against *S. aureus* and 26 and 52 against *E. coli*, respectively. It is interesting to note that TC-loaded CMC-Cu-MEL and TC-loaded CMC-Zn-MEL were both even more effective than ampicillin against *S. aureus*. According to these findings, TC is released from TC-loaded CMC-Cu-MEL and TC-loaded CMC-Zn-MEL, and is effective against *E. coli* and *S. aureus*.

## 4. Conclusions

Hydrothermal preparation of CMC-Cu-MEL and CMC-Zn-MEL, two drug delivery systems based on carboxymethyl cellulose with copper oxide at melamine and zinc oxide at melamine framework, was successful. FTIR, SEM, Raman spectroscopy, TEM, and SAED were used to characterize synthesized nanocomposites. Additionally, using XRD, the crystallinity of the produced materials was investigated. The nitrogen adsorption–desorption isotherm was used to determine the total surface area and particle size distribution of the prepared nanocomposites. The data revealed that the scale size was found to be between 5 and 25 nm, which confirms the nanoscale-size of the prepared CMC-Cu-MEL and CMC-Zn-MEL materials. Furthermore, SAED images of CMC-Cu-MEL and CMC-Zn-MEL have different ring patterns with variable diameters, which are assigned to different planes of the formed crystals. Additionally, SAED affirms the design of material in nanocrystalline characteristics. XRD analysis confirmed the crystallinity of synthesized CMC-Cu-MEL and CMC-Zn-MEL materials with higher intensity peaks at 35.26, and 38.66°, which indicate the formation of CuO, and 2θ peaks were observed at 31.5, 34.18, 36.02, 47.32, 56.31, 62.66, and 67.64°, which indicate ZnO formation. In addition, the BET surface area of the CMC, CMC-Cu-MEL, and CMC-Zn-MEL materials were calculated and found at 64.4218, 5.8830, and 5.3964 m^2^/g, respectively. Therefore, the introduction of metallic parts decreased the estimated BET surface area. In addition, there was an increase in drug release within the first two hours, which is mostly attributable to the high charge density and diffusion-controlled release. Additionally, the Ritger–Peppas kinetic model’s assessment of exponent n as the transport factor with a predominately solvent diffusion-related effect elaborated the diffusion and polymeric relaxation processes in the context of the drug release mechanisms of TC over CMC-Cu-MEL and CMC-Zn-MEL. The highest antibacterial effects, however, were demonstrated by the synthetic TC-loaded CMC-Cu-MEL and TC-loaded CMC-Zn-MEL, which had activity indices of 99 and 106 percent against *S. aureus* and 93 and 99 percent against *E. coli*, respectively. The synthetic CMC-Cu-MEL and CMC-Zn-MEL, on the other hand, demonstrated very weak antibacterial activity, with activity index percentages of 34 and 66 against *S. aureus* and 26 and 52 against *E. coli*, respectively. Additionally, TC-loaded CMC-Cu-MEL and TC-loaded CMC-Zn-MEL nanocomposites were toxic to both bacteria.

## Figures and Tables

**Table 1:** The antibacterial activity of the TC-loaded CMC-Cu-MEL and TC-loaded CMC-Zn-MEL.

Samples	*E. coli*		*S. aureus*	
	Diameter (mm) ^a^	% Activity Index	Diameter (mm) ^a^	% Activity Index
CMC-Cu-MEL	6	26	8.8	34
CMC-Zn-MEL	12	52	17.2	66
TC-loaded CMC-Cu-MEL	21.4	93	25.8	99
TC-loaded CMC-Zn-MEL	22.8	99	27.6	106
Ampicillin ^b^	23	-	26	-

^a^ Disk papers (5 mm) were soaked with 20 μM of the suspended solution of prepared nanocomposites in DMSO. ^b^ The activity was measured after one-day incubation with the prepared nanocomposites. Ampicillin was used as the reference. All experiments were done in replicates.

## Data Availability

The raw/processed data generated in this work are available upon request from the corresponding author.

## Supplementary Materials

The following supporting information can be downloaded at: https://www.mdpi.com/article/10.3390/polym14183815/s1, Figure S1: Antibacterial activity of TC-loaded-CMC-Cu-MEL and TC-loaded-CMC-Zn-MEL, +Ve control is the ampicillin.
