# Supplementary material for: Drug Delivery System Based on Carboxymethyl Cellulose Containing Metal-Organic Framework and Its Evaluation for Antibacterial Activity
Source: Polymers (Basel). 2022 Sep 12;14(18):3815. doi: 10.3390/polym14183815 (PMC9503747; doi:10.3390/polym14183815)
Supplement: Supplementary file 1 [file polymers-14-03815-s001.zip › polymers-1895350-supplementary.pdf]

Drug Delivery System Based on Carboxymethyl Cellulose Containing Metal-organic Framework and Their Evaluation as Antibacterial Activity

Fatimah A.T. Alsaeed [1], Hany M. Abd El-Lateef [1,2,*], Mai M. Khalaf [1,2], Ibrahim M. A. Mohamed [2,*], M. A. Al-Omair [1] and Mohamed Gouda [1,*]

[1] Department of Chemistry, College of Science, King Faisal university, P.O. Box 400 Al-Ahsa 31982, Saudi Arabia
[2] Chemistry Department, Faculty of Science, Sohag University, Sohag 82524, Egypt
* Correspondence: hmahmed@kfu.edu.sa, hany_shubra@science.sohag.edu.eg (H.M Abd El-Lateef), ibrahim_mohamed@science.sohag.edu.eg (Ibrahim M. A. Mohamed), mgoudaam@kfu.edu.sa (M. Gouda)

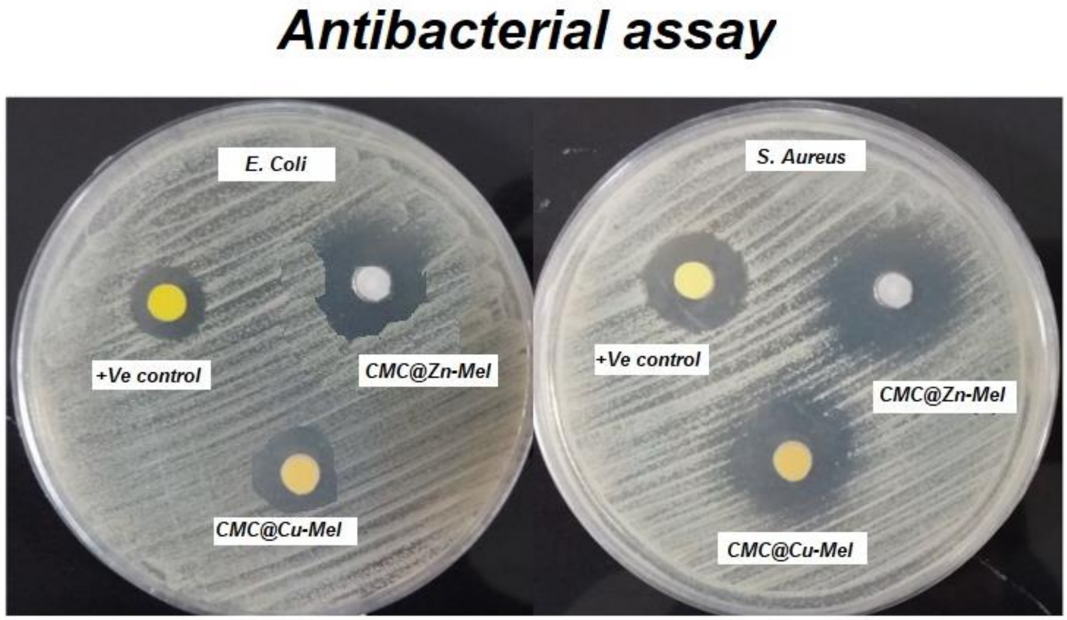

Figure S1. Antibacterial activity of TC-loaded-CMC-Cu-MEL, and TC-loaded-CMC-Zn-MEL

+Ve control is the ampicillin
